# Substitutional and interstitial impurity p-type doping of thermoelectric Mg_2_Si: a theoretical study

**DOI:** 10.1080/14686996.2019.1580537

**Published:** 2019-03-14

**Authors:** Naomi Hirayama, Tsutomu Iida, Mariko Sakamoto, Keishi Nishio, Noriaki Hamada

**Affiliations:** a Graduate School of Science, Osaka University, Toyonaka, Osaka, Japan; b Faculty of Industrial Science and Technology, Tokyo University of Science, Tokyo, Japan; c Center for Spintronics Research Network, Osaka University, Toyonaka, Osaka, Japan; d Faculty of Science and Technology, Tokyo University of Science, Noda, Chiba, Japan

**Keywords:** Magnesium silicide, hole doping, thermoelectric properties, interstitial insertion, p-type semiconductor, structural stability, 50 Energy Materials, 210 Thermoelectronics / Thermal transport / insulators

## Abstract

The narrow-gap magnesium silicide semiconductor Mg_2_Si is a promising mid-temperature (600–900 K) thermoelectric material. It intrinsically possesses n-type conductivity, and n-type dopants are generally used for improving its thermoelectric performance; however, the synthesis of p-type Mg_2_Si is relatively difficult. In this work, the hole doping of Mg_2_Si with various impurity atoms is investigated by performing first principles calculations. It is found that the Ag-doped systems exhibit comparable formation energies Δ*E* calculated for different impurity sites (Mg, Si, and interstitial 4b ones), which may explain the experimental instability of their p-type conductivity. A similar phenomenon is observed for the systems incorporating alkali metals (Li, Na, and K) since their Δ*E* values determined for Mg (p-type) and 4b (n-type) sites are very close. Among boron group elements (Ga and B), Ga is found to be favorable for hole doping because it exhibits relatively small Δ*E* values for Si (p-type) sites. Furthermore, the interstitial insertion of Cl and F atoms into the crystal lattice leads to hole doping because of their high electronegativity.

## Graphical abstract

**Figure:**
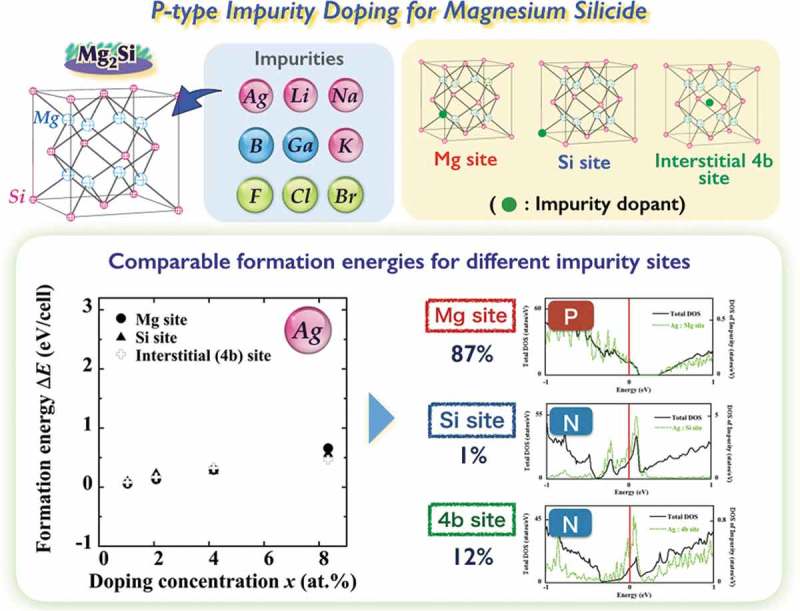

## 1. Introduction

Thermoelectric generators that directly convert thermal energy into electric energy through the Seebeck effect can be potentially used for recovering waste heat energy to improve the energy efficiency of multiple applications, thus providing a possible solution to various environmental problems, such as global warming and limited energy resources.

Thermoelectric silicide materials have attracted considerable attention over the last decade because of their potential applicability in renewable and sustainable energy technologies. Magnesium silicide (Mg_2_Si) [1–3], a narrow-gap semiconductor with anti-fluorite crystal structure, is a promising candidate for mid-temperature (600–900 K) thermoelectric applications, owing to its non-toxicity, low production cost, and low weight. However, significant improvement of the thermoelectric conversion efficiency of Mg_2_Si is required for its successful commercialization.

N-type dopants such as Sb [1], Al [4], Bi [4], and Sn [5] were previously utilized to tune the carrier concentration in the Mg_2_Si crystal and improve its thermoelectric efficiency. However, despite the multiple attempts to locate suitable acceptors, the development of hole-doped Mg_2_Si is currently hindered by the difficulties in achieving stable p-type conductivity. For example, according to the results of one experimental study [6], the Ag doping of Mg_2_Si results in p-type conductivity; however, the obtained systems exhibit the change in conductivity from p-type to n-type at temperatures above 650 K. This instability of p-type conductivity represents one of the obstacles to the successful development of Mg_2_Si-based thermoelectric power generators because the conventional thermoelectric modules have a π-shape structure consisting of both n-type and p-type semiconductors. Therefore, the realization of a stable p-type Mg_2_Si semiconductor with high thermoelectric efficiency would significantly contribute to the development of thermoelectric power generators.

As indicated by the results of theoretical [7,8] and experimental [9] studies, the existence of native lattice defects strongly affects the carrier transport in the Mg_2_Si structure. In particular, the Mg atoms interstitially inserted into the cell act as dopants, owing to the intrinsic n-type conductivity of Mg_2_Si [7]. A previous theoretical study [10] suggested that in the systems containing both lattice defects and acceptors, the number of electrons generated by interstitial Mg doping could compensate for the number of holes produced by intentional impurity doping and even potentially exceed it if a system contained interstitial Mg defects with a comparable number of acceptors. Thus, the presence of interstitial Mg defects is one of the main reasons for the difficulty in achieving stable p-type conductivity of Mg_2_Si.

In the present study, we focus on another possible origin of the observed instability of p-type Mg_2_Si – the structural instability of impurity atoms. By today, many theoretical studies [11–17] have been performed to determine the electronic properties of impurity-doped Mg_2_Si and select the most suitable acceptors for this system. However, detailed and comprehensive information regarding the site occupation and stability of impurities in a cell has not been obtained yet. According to the results of this work, Ag atoms can occupy not only p-type (Mg), but also n-type (Si and interstitial) sites because of the small differences in their formation energies. Furthermore, it is suggested that Ag atoms change their locations at operating temperatures (600–900 K), which is presumably related to the above-mentioned variation of the conduction type [6]. In addition, Li [15], Na [16], and K alkaline metals, as well as B and Ga [18] boron group elements are found to generate holes after doping the most stable sites; however, except for B, they can dope different sites simultaneously because of their close formation energies. Furthermore, the feasibility of achieving p-type conductivity through the interstitial insertion of impurities characterized by high electronegativity (F, Cl, and Br) has been evaluated.

## 2. Computational method

### 2.1. Structural optimization of impurity-doped Mg_2_Si crystals

**Figure 1.**
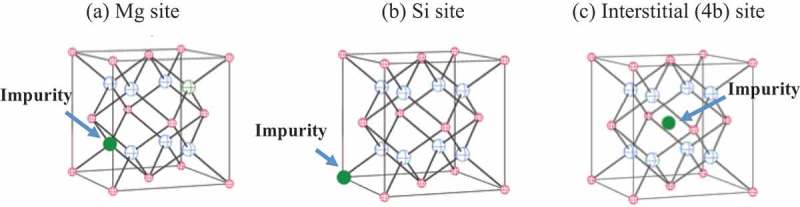
A Mg_2_Si crystal unit cell (Fm-3m space group) consisting of the Si and Mg atoms denoted by the small and large spheres at the 4a and 8c sites, respectively. Possible impurity sites: (a) Mg atom, (b) Si atom, and (c) 4b site.

Mg_2_Si has the anti-fluorite crystal structure that belongs to the Fm-3m space group (No. 225). Its Si and Mg atoms occupy the 4a and 8c sites, respectively, and their atomic positions in the conventional unit cell (depicted in Figure 1) correspond to the following coordinates: Si (0, 0, 0), Mg (0.25, 0.25, 0.25), and Mg (0.75, 0.75, 0.75). Figure 1 describes the three impurity-doped systems examined in the present study: the substitution of an impurity atom for the Si and Mg atoms and its interstitial insertion into the 4b site (0.5, 0.5, 0.5).

The impurity doping concentration *x* (at.%) is defined as its atomic fraction calculated with respect to the total number of atoms in the cell. As shown in Figure 1, the Mg_2_Si unit cell contains 12 (4 Si and 8 Mg) atoms; thus, assuming simple cubic (sc) periodicity for the atomic positions of impurities, their fraction in the cell *x* is equal to 8.333 at.% (1 impurity per 12 atoms). In order to examine the impurity-doped systems with *x* values close to the experimental ones (1 at.% or less), a 2×2×2 supercell that consists of eight unit cells with 96 (32 Si and 64 Mg) atoms must be utilized. When one atom (Mg or Si) in the 2×2×2 supercell is replaced by one (or two) impurity atoms with the sc periodicity, the value of *x* becomes equal to 1.042 at.% (or 2.083 at.%). Similarly, when the impurity distribution follows the fcc periodicity, a total of four impurity atoms are introduced into the supercell containing 96 atoms, which results in *x* = 4.167 at.%.

The obtained structures of the impurity-doped systems were optimized by taking into account the localized shifts of individual atoms from their regular positions in the perfect crystal caused by the presence of impurity atoms, as well as by optimizing the lattice constants using the cell-relaxation technique of the Quantum Espresso package [19] (which included a pseudopotential method based on the density functional theory). In all calculations, the norm-conserving pseudopotentials and generalized gradient approximation method (represented by the Perdew–Burke–Ernzerhof functional) were utilized. Wave functions, charge density, and potential were expanded into the plane waves with the cutoff parameters equal to 60 Ry for the wave functions and 240 Ry for the charge densities and potentials. Brillouin zone sampling was performed using the ***k***-point grid with the 8×8×8 FCC mesh. For self-consistent field calculations, the convergence threshold of the total energy was equal to 10^–8^ Ry. A variable cell relaxation procedure was performed using the Broyden–Fletcher–Goldfarb–Shanno quasi-newton algorithm with the convergence thresholds for the total energy, all force components, and cell pressure equal to 10^–5^ Ry, 10^–4^ Ry/Bohr, and 0.5 kbar, respectively.

### 2.2. Formation energies and distributions of impurities

In order to identify the preferred occupation sites for various impurities in terms of energy, the following formation energies were calculated for the optimized structures:

(1) $$\Delta E^{Mg\;site}=E\left(Mg_{2-3x}SiA_{3x}\right)+3xE\left(Mg\right)-E\left(Mg_{2}Si\right)-3xE\left(A\right),$$

(2) $$\Delta E^{Si\;site}=E\left(Mg_{2}Si_{1-3x}A_{3x}\right)+3xE\left(Si\right)-E\left(Mg_{2}Si\right)-3xE\left(A\right),$$

(3) $$\Delta E^{4b\;site}=E\left(Mg_{2}SiA_{3x}\right)-E\left(Mg_{2}Si\right)-3xE\left(A\right).$$

Equations (1)–(3) correspond to the Mg, Si, and 4b sites occupied by impurity atoms A (see Figure 1) (here E denotes the total energy values calculated assuming single-crystal structures for the studied systems). Although B can assume several crystal configurations, the crystallographic data for its rhombohedral lattice [20] were used to estimate the E(B) values. In addition, CaF_2_ [21] and crystalline Cl_2_ [22] were also considered precursors for doping the Mg_2_Si system with F and Cl atoms at a temperature of 88.15 K, respectively, because they exist in the gaseous state at normal pressure and room temperature. Hence, Equations (1)–(3) for the CaF_2_ dopant can be expressed the following forms:

(1) $$\Delta E_{Mg\;site}=E\left(Mg_{2-3x}SiF_{3x}\right)+\frac{3x}{2}E\left(Ca\right)+3xE\left(Mg\right)-E\left(Mg_{2}Si\right)-\frac{3x}{2}E\left(CaF_{2}\right),$$

(2) $$\Delta E_{Si\;site}=E\left(Mg_{2}Si_{1-3x}F_{3x}\right)+\frac{3x}{2}E\left(Ca\right)+3xE\left(Si\right)-E\left(Mg_{2}Si\right)-\frac{3x}{2}E\left(CaF_{2}\right),$$

(3) $$\Delta E_{4b\;site}=E\left(Mg_{2}SiF_{3x}\right)+\frac{3x}{2}E\left(Ca\right)-E\left(Mg_{2}Si\right)-\frac{3x}{2}E\left(CaF_{2}\right).$$

If an impurity-doped system exhibits close formation energies for different sites, both its n-type and p-type sites can be occupied during synthesis, which prevents the realization of well-controlled carrier doping. To estimate the site preferences of the utilized dopants quantitatively, the values of the occupation probability of an impurity atom *p_i_* were calculated for Mg, Si, and interstitial 4b sites. During calculations, it was assumed that impurity atoms were distributed across different sites as a canonical ensemble; therefore, the value of *p_i_* was proportional to the Boltzmann factor $exp\left(-\beta E_{i}\right)$, where $E_{i}$ was the energy required for occupying site *i*. Further, impurity atoms were also considered independent from each other; in this case, the formation energy per impurity atom $\Delta \varepsilon_{i}$ was equal to $E_{i}$. Hence,

(4) $$p_{Mg}\;=\;2\;Z^{-1}exp\left(-\frac{\Delta \varepsilon_{Mg\;site}}{k_{B}T}\right),$$

(5) $$p_{i}\;=\;Z^{-1}exp\left(-\frac{\Delta \varepsilon_{i}}{k_{B}T}\right),\;i=Si\;and\;4b\;sites$$

where $k_{B}$ is the Boltzmann constant, and the partition function *Z* is defined as:

(6) $$Z\;=\;2exp\left(-\frac{\Delta \varepsilon_{Mg\;site}}{k_{B}T}\right)+exp\left(-\frac{\Delta \varepsilon_{Si\;site}}{k_{B}T}\right)+exp\left(-\frac{\Delta \varepsilon_{4b\;site}}{k_{B}T}\right).$$

The temperature used in the synthesis by the vertical Bridgman technique (1378 K [23]) was substituted into *T* of Equations (4)–(6). The obtained *p_i_* values were used to predict whether the added impurities would occupy not only the p-type sites, but also the n-type ones during synthesis.

## 3. Results and discussion

### 3.1. Site stability of conventional p-type Ag dopant

In this section, the stability of the sites occupied by Ag atoms in the Mg_2_Si crystal lattice (which represent typical p-type dopants) is discussed in terms of the formation energies ΔE of the optimized Ag-doped structures.

Table 1 lists the changes in the lattice constant *a* from that of pure Mg_2_Si (6.354 Å) caused by doping with Ag atoms and the positions of its neighboring atoms determined via the variable cell relaxation calculations. Although this table contains only the data obtained for the 2×2×2 cell incorporating an Ag atom, the values of *a* determined at other doping concentrations *x* are presented in Supplemental Material 1. These results show that the largest lattice constant was obtained for doping the 4b sites followed by the Si and Mg sites. In the first case, the insertion of an additional atom into the cell increased its lattice constant. In fact, the analysis of atomic positions revealed that when an Ag atom was inserted into the 4b site, the surrounding Mg atoms moved away from it by a relative distance of 2.6%. During substitutional doping, the crystal is expected to expand (or shrink) after the replacement of a Si (Mg) atom with Ag because the latter has a greater (lower) covalent radius (126 pm) as compared to 116 pm for Si and 139 pm for Mg [24]. During the substitution of Si with Ag, the atomic positions remain mostly unchanged. In the case of Mg sites, the introduced Ag atom attracts the Mg atoms occupying the next-nearest neighbor sites causing them to move at a relative distance of 3.4%, which is larger than the displacement of the nearest Si atoms. Hence, it can be concluded that the observed shrinkage of the cell should be attributed to the interactions between the Ag species and their next-nearest Mg neighbors (in other words, the Ag atoms substituted for Mg ones occupy a smaller volume because of their smaller covalent radii; as a result, the next-neighboring Mg atoms move towards Ag ones).

**Table 1.** Lattice parameters and coordinates of individual atoms in the 2×2×2 supercell (in lattice parameter units) containing 1.04% of impurities obtained via variable cell relaxation calculations. The regular positions of the atoms in the perfect crystal are listed as well. The lattice parameters are shown as changes from that of the perfect crystal, which is equal to 6.357 Å for the conventional unit cell. The term Δr denotes the calculated changes in the distances from the impurity positions to their neighboring atoms.

Impurity	Occupation site*	Lattice parameter (**)	*i*-th nearest atom	Regular position	Optimized position	Δr (%)
Ag	Mg site	6.348 Å	*i* = 1 (Si)	(0 0 0)	(−0.0014 −0.0014 −0.0014)	1.1
		(−0.094%)	*i *= 2 (Mg)	(0.125 0.125 0.375)	(0.125 0.125 0.3664)	−3.4
	Si site	6.358 Å	*i *= 1 (Mg)	(0.125 0.125 0.125)	(0.1245 0.1245 0.1245)	−0.4
		(+0.063%)	*i *= 2 (Si)	(0 0.25 0.25)	(0 0.2495 0.2495)	−0.2
	4b site	6.359 Å	*i *= 1 (Mg)	(0.125 0.125 0.125)	(0.1217 0.1217 0.1217)	2.6
		(+0.079%)	*i *= 2 (Si)	(0 0.25 0.25)	(0.0007 0.25 0.25)	−0.3
Li	Mg site	6.348 Å	*i* = 1 (Si)	(0 0 0)	(−0.0001 −0.0001 −0.0001)	0.1
		(−0.094%)	*i *= 2 (Mg)	(0.125 0.125 0.375)	(0.125 0.125 0.368)	−2.8
	Si site	6.357 Å	*i *= 1 (Mg)	(0.125 0.125 0.125)	(0.1248 0.1248 0.1248)	−0.2
		(+0.047%)	*i *= 2 (Si)	(0 0.25 0.25)	(0 0.2494 0.2494)	−0.2
	4b site	6.353 Å	*i *= 1 (Mg)	(0.125 0.125 0.125)	(0.1215 0.1215 0.1215)	2.8
		(−0.016%)	*i *= 2 (Si)	(0 0.25 0.25)	(0.0053 0.25 0.25)	−2.1
Na	Mg site	6.358 Å	*i* = 1 (Si)	(0 0 0)	(−0.0037 −0.0037 −0.0037)	2.9
		(+0.063%)	*i *= 2 (Mg)	(0.125 0.125 0.375)	(0.125 0.125 0.3711)	−1.6
	Si site	6.369 Å	*i *= 1 (Mg)	(0.125 0.125 0.125)	(0.1336 0.1336 0.1336)	6.9
		(+0.236%)	*i *= 2 (Si)	(0 0.25 0.25)	(0 0.2503 0.2503)	0.2
	4b site	6.362 Å	*i *= 1 (Mg)	(0.125 0.125 0.125)	(0.1192 0.1192 0.1192)	4.6
		(+0.126%)	*i *= 2 (Si)	(0 0.25 0.25)	(0.0040 0.25 0.25)	−1.6
K	Mg site	6.370 Å	*i* = 1 (Si)	(0 0 0)	(−0.0075 −0.0075 −0.0075)	6.0
		(+0.252%)	*i *= 2 (Mg)	(0.125 0.125 0.375)	(0.125 0.125 0.3757)	0.3
	Si site	6.386 Å	*i *= 1 (Mg)	(0.125 0.125 0.125)	(0.145 0.145 0.145)	16
		(+0.504%)	*i *= 2 (Si)	(0 0.25 0.25)	(0 0.2513 0.2513)	0.5
	4b site	6.373 Å	*i *= 1 (Mg)	(0.125 0.125 0.125)	(0.1169 0.1169 0.1169)	6.5
		(+0.299%)	*i *= 2 (Si)	(0 0.25 0.25)	(0.0021 0.25 0.25)	−0.8
B	Mg site	6.332 Å	*i* = 1 (Si)	(0 0 0)	(0.0179 0.0179 0.0179)	−14
		(−0.346%)	*i *= 2 (Mg)	(0.125 0.125 0.375)	(0.125 0.125 0.3757)	0.3
	Si site	6.334 Å	*i *= 1 (Mg)	(0.125 0.125 0.125)	(0.1159 0.1159 0.1159)	−7.3
		(−0.315%)	*i *= 2 (Si)	(0 0.25 0.25)	(0 0.2464 0.2464)	−1.4
	4b site	6.347 Å	*i *= 1 (Mg)	(0.125 0.125 0.125)	(0.1264 0.1264 0.1264)	−1.1
		(−0.110%)	*i *= 2 (Si)	(0 0.25 0.25)	(0.0013 0.25 0.25)	−0.5
Ga	Mg site	6.346 Å	*i* = 1 (Si)	(0 0 0)	(0.0033 0.0033 0.0033)	−2.6
		(−0.126%)	*i *= 2 (Mg)	(0.125 0.125 0.375)	(0.125 0.125 0.3732)	−0.7
	Si site	6.355 Å	*i *= 1 (Mg)	(0.125 0.125 0.125)	(0.1236 0.1236 0.1236)	−1.1
		(+0.016%)	*i *= 2 (Si)	(0 0.25 0.25)	(0 0.2494 0.2494)	−0.2
	4b site	6.359 Å	*i *= 1 (Mg)	(0.125 0.125 0.125)	(0.1208 0.1208 0.1208)	3.4
		(+0.079%)	*i *= 2 (Si)	(0 0.25 0.25)	(0.0026 0.25 0.25)	−1.0
F	Mg site	6.337 Å	*i* = 1 (Si)	(0 0 0)	(−0.0132 −0.0132 −0.0132)	11
		(−0.268%)	*i *= 2 (Mg)	(0.125 0.125 0.375)	(0.125 0.125 0.3333)	−17
	Si site	6.325 Å	*i *= 1 (Mg)	(0.125 0.125 0.125)	(0.1250 0.1250 0.1250)	0.0
		(−0.456%)	*i *= 2 (Si)	(0 0.25 0.25)	(0 0.2461 0.2461)	−1.6
	4b site	6.336 Å	*i *= 1 (Mg)	(0.125 0.125 0.125)	(0.1298 0.1298 0.1298)	−3.8
		(−0.283%)	*i *= 2 (Si)	(0 0.25 0.25)	(−0.0029 0.25 0.25)	−1.2
Cl	Mg site	6.363 Å	*i* = 1 (Si)	(0 0 0)	(−0.0128 −0.0128 −0.0128)	10
		(+0.142%)	*i *= 2 (Mg)	(0.125 0.125 0.375)	(0.125 0.125 0.3581)	−6.8
	Si site	6.358 Å	*i *= 1 (Mg)	(0.125 0.125 0.125)	(0.1339 0.1339 0.1339)	7.1
		(+0.063%)	*i *= 2 (Si)	(0 0.25 0.25)	(0 0.2485 0.2485)	−0.6
	4b site	6.368 Å	*i *= 1 (Mg)	(0.125 0.125 0.125)	(0.1249 0.1249 0.1249)	0.1
		(+0.220%)	*i *= 2 (Si)	(0 0.25 0.25)	(−0.0064 0.25 0.25)	2.6
Br	Mg site	6.367 Å	*i* = 1 (Si)	(0 0 0)	(−0.0127 −0.0127 −0.0127)	10
		(+0.205%)	*i *= 2 (Mg)	(0.125 0.125 0.375)	(0.1246 0.1246 0.3649)	−4.0
	Si site	6.364 Å	*i *= 1 (Mg)	(0.125 0.125 0.125)	(0.1368 0.1368 0.1368)	2.0
		(+0.157%)	*i *= 2 (Si)	(0 0.25 0.25)	(0 0.2491 0.2491)	−0.4
	4b site	6.377 Å	*i *= 1 (Mg)	(0.125 0.125 0.125)	(0.1221 0.1221 0.1221)	2.3
		(+0.362%)	*i *= 2 (Si)	(0 0.25 0.25)	(−0.0008 0.2495 0.2495)	0.3

*Positions of the impurity atoms were set to the values obtained before performing variable cell relaxation calculations (corresponding to the regular positions of the atoms in the 2×2×2 super cell of the perfect crystal): Mg (0.125, 0.125, 0.125), Si (0, 0, 0), and 4b (0.25, 0.25, 0.25). **Changes in lattice parameter compared to the perfect crystal (*a *= 6.354 Å) are shown in the parenthesis.

**Figure 2.**
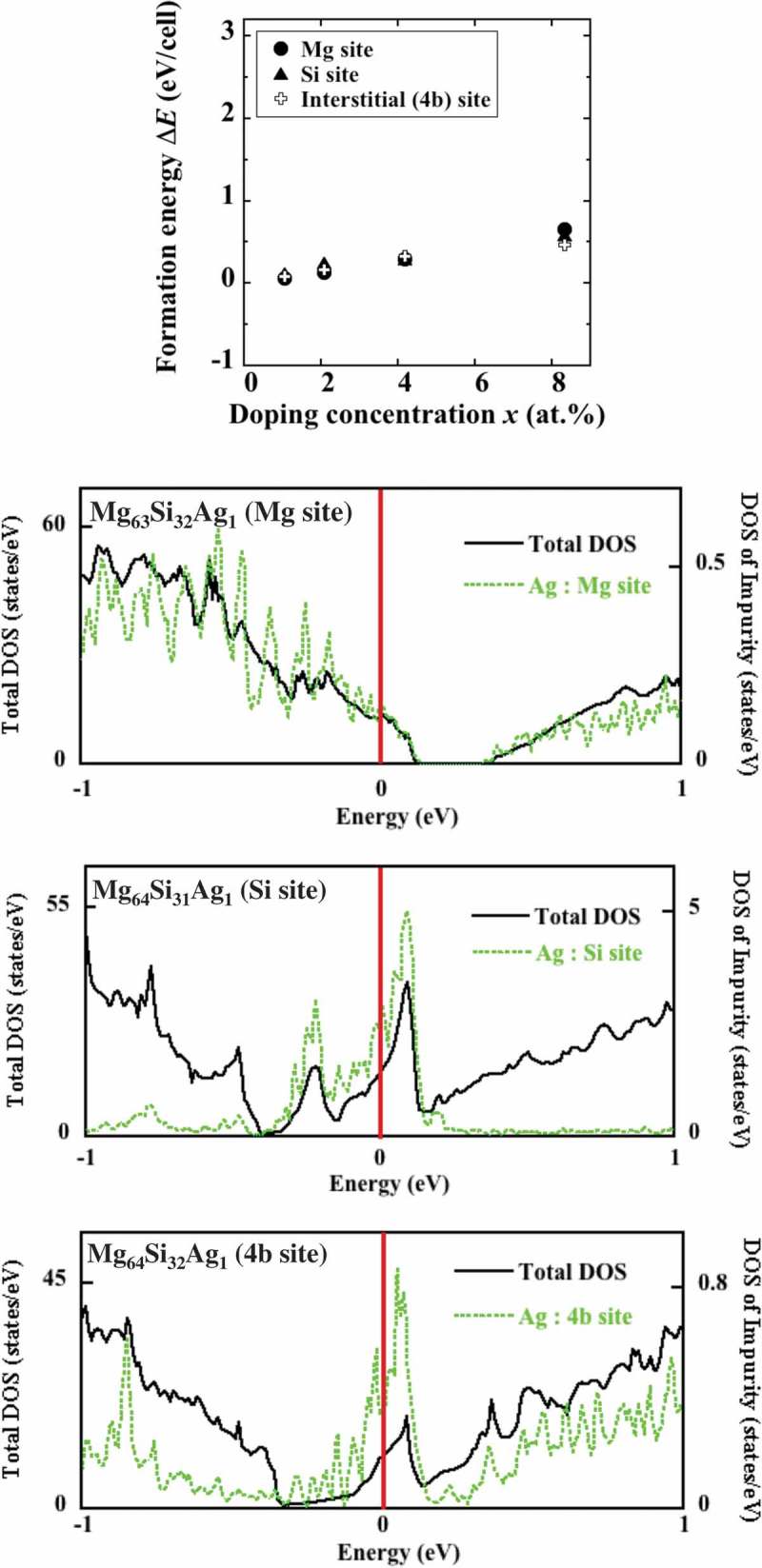
(Top) Dependences of the formation energy of Ag-doped Mg_2_Si on the doping concentration determined for Mg, Si, and 4b sites. Because the formation energies obtained at each concentration are relatively close, Mg doping may be interchanged with Si or 4b doping at a small difference in energy. (Bottom) The DOS of the crystals doped with Ag atoms occupying the Mg, Si, and 4b sites at *x *= 1.04 at.%. The Mg sites exhibit p-type conductivity, while the other two sites (Si and 4b) are characterized by n-type conductivity.

**Figure 3.**
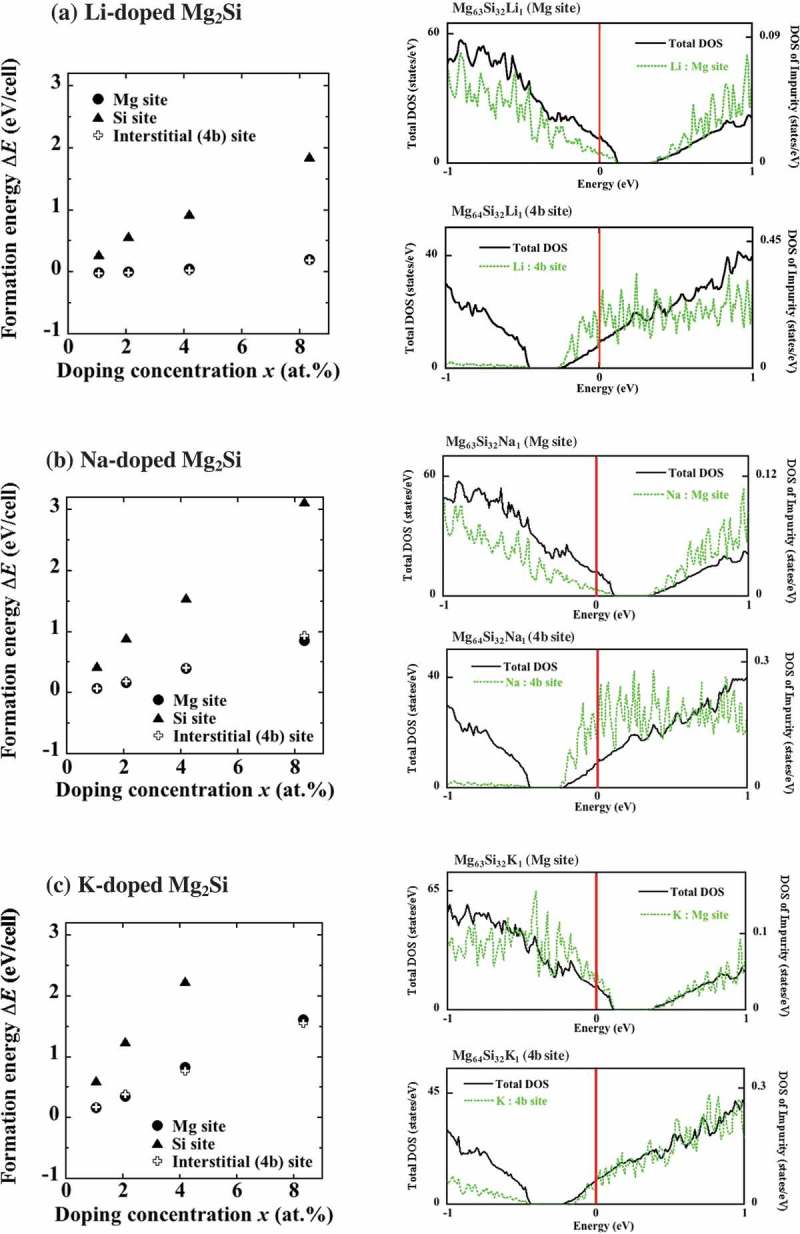
Formation energies and DOS values of (a) Li-, (b) Na-, and (c) K-doped Mg_2_Si determined for Mg (circles), Si (triangles), and 4b (crosses) sites. Here, the results of ΔE(*x*) for Na-doped systems are almost identical to ones obtained in our previous study [25] where the different calculation conditions were used. The obtained results show that Mg and 4b sites are the two preferred sites for these impurities. The DOS of these systems at the concentration *x *= 1.04 at.% demonstrate that the substitution of Mg atoms with these impurities generated holes, whereas the occupation of 4b sites led to electron doping.

**Figure 4.**
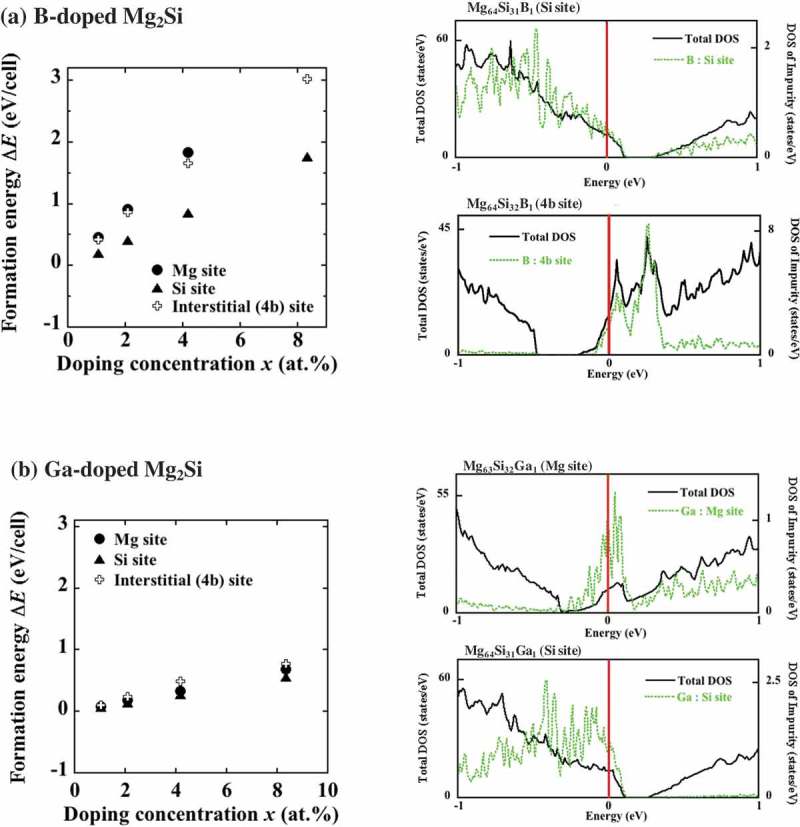
Formation energies ΔE(*x*) and DOS values of (a) B- and (b) Ga-doped Mg_2_Si for Mg (circles), Si (triangles), and 4b (crosses) sites. Here, the ΔE(*x*) for B-doped systems are similar to the calculation results obtained under different condition [25]. According to the obtained formation energies, both impurities tend to substitute Si atoms. The DOS of these systems at the concentration *x *= 1.04 at.% exhibit p-type conductivity when the impurities occupy the most stable Si sites.

**Figure 5.**
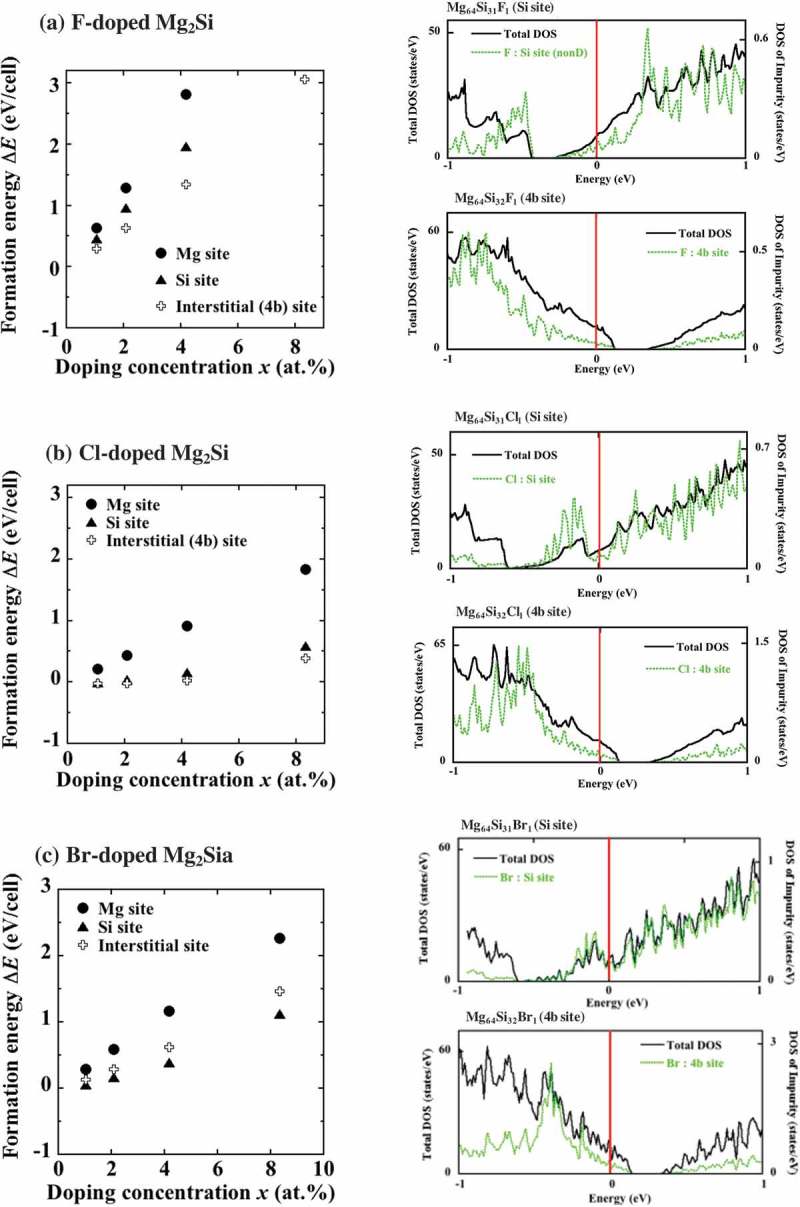
Formation energies and DOS of (a) F-, (b) Cl-, and (c) Br-doped Mg_2_Si determined for Mg (circles), Si (triangles), and 4b (crosses) sites. The F and Cl atoms occupying 4b sites exhibit the minimum formation energies. The DOS values of the Mg_2_Si crystals doped with (g) F, (h) Cl, and (i) Br atoms at the concentration *x *= 1.04 at.% show that these systems exhibit p-type conductivity for the 4b sites.

The formation energies of the optimized structures $\Delta E_{i}$ (*i *= Mg, Si, or 4b) were calculated using Equations (1)–(3). Figure 2 shows the dependences of $\Delta E^{i}$ on *x*. The numerical error of each data point was below 10^–10^ Ry in this and the subsequent figures, in which the error bars are not shown because they fall within the areas covered by the symbols. It should be noted that Figure 2 and the subsequent Figures 3–5 display the formation energies $\Delta E_{i}$that have been converted to the values obtained for the conventional unit cell depicted in Figure 1. On the other hand, to calculate the occupational probabilities $\;p_{i}$ using Equations (4)–(6), the formation energies per impurity atom $\Delta \varepsilon_{i}$ are used, which are explained in more detail in the Supplemental Material 2.

According to Figure 2, the studied systems exhibit close values of $\Delta E_{i}\;\left(i=Mg,Si,\;or\;4b\right)$; therefore, doping different sites with Ag can be achieved during the synthesis procedure. The densities of states (DOS) of the Mg_2_Si crystals doped with Ag atoms at the concentration *x *= 1.04 at.% (1 impurity per 96 atoms in a cell) are also depicted in Figure 2. Their values indicate that for the Mg sites, the position of the Fermi energy level shifts towards the valence band leading to p-type conductivity, while for the other cases, it shifts towards the conduction band, leading to n-type conductivity.

The$\;p_{i}\;\left(i=Mg,Si,or\;\;4b\right)$values calculated using Equations (4)–(6) are listed in Table 2; $\Delta \varepsilon_{i}$ is obtained by fitting the $\Delta E_{i}\left(x\right)$ data plotted in Figure 2 using the method of least squares (here the values of *x* do not exceed 4.17% because their experimental values are typically equal to 1% or less). The computational details are provided in Supplemental Material 2. As a result, the values $\Delta \varepsilon_{Mg\;site}$ = 0.525 eV, $\Delta \varepsilon_{Si\;site}$ = 1.0 eV, $\Delta \varepsilon_{4b\;site}$= 0.675 eV, and accordingly, $p_{Mg}=0.87,\;p_{Si}=0.01,$ and $p_{4b}=0.12$ were obtained at a synthesis temperature of 1378 K, suggesting that the added Ag atoms mainly replaced Mg ones to generate holes; however, some of them occupied other sites (especially 4b ones) to produce electrons. Nevertheless, the resulting system should exhibit p-type conductivity because the majority of Ag atoms occupied the Mg sites.

**Table 2.** Formation energies per impurity atom $\Delta \varepsilon_{i}$ (*i* = Mg, Si, or 4b), which were obtained using the procedure described in Supplemental Material 2, and the corresponding occupation probabilities of impurity sites $p_{i}$ determined by Equations (4)–(6) .

Impurity	Occupation site	Conductivity type	Formation energy per atom Δε (eV)	Site occupation probability* (%)
Ag	Mg site	p	0.525	87
	Si site	n	1.000	1
	4b site	n	0.675	12
Li	Mg site	p	−0.008	55
	Si site	n	2.283	0
	4b site	n	−0.067	45
Na	Mg site	p	0.633	82
	Si site	n	3.592	0
	4b site	n	0.733	18
K	Mg site	p	1.400	88
	Si site	n	4.992	0
	4b site	n	1.550	12
B	Mg site	n	3.642	0
	Si site	p	1.650	100
	4b site	n	3.442	0
Ga	Mg site	n	0.719	30
	Si site	p	0.540	68
	4b site	n	0.982	2
F	Mg site	p	5.142	0
	Si site	n	3.800	0
	4b site	P	2.508	100
Cl	Mg site	p	1.733	0
	Si site	n	0.125	14
	4b site	p	−0.092	86
Br	Mg site	p	2.350	0
	Si site	n	0.660	98
	4b site	p	1.150	2

*Site occupation probability is calculated at synthesis temperature (1378 K).

In the discussion presented above, the multiple-site occupation with Ag atoms that occurs during synthesis was considered; however, a change in the type of the occupation site likely occurs when a thermoelectric generator is operated at elevated temperatures because of the very small energetic differences between the sites, as shown in Figure 2. The difference between $\Delta \varepsilon_{Mg\;site}$ and $\Delta \varepsilon_{4b\;site}$ is about 0.15 eV, which is relatively close to the heat energies of 0.05–0.08 eV corresponding to the temperature range of 600–900 K (note that Mg_2_Si-based materials are expected to be used in this temperature range). Therefore, it may be possible that the Ag atoms occupying the Mg sites move to the 4b sites when the system is operated at high temperatures characterized by the dominant entropic contribution to the free energy. The heat-induced change in the type of occupation sites may explain the experimental findings [6] indicating that the conduction of Ag-doped Mg_2_Si changes from p-type to n-type at temperatures above 650 K.

Additionally, if the Mg_2_Si cell expands due to the applied heat, it can also increase the thermal instability of p-type conductivity because the optimized cell obtained for the p-type (Mg) site shrinks by doping with Ag, in contrast to the other sites. Thus, the Ag atoms substituted for Mg can become more unstable due to thermal expansion, which may further reduce the number of holes at elevated temperatures.

Furthermore, the site occupation of Ag may be controlled by applying an external pressure during synthesis. To estimate the pressure-induced shift of $\Delta \varepsilon_{i}$, we calculated the electronic state for systems containing Ag atoms at 1.04 at.%, which occupy the n-type (Si or 4b) sites, with the lattice constant of the Mg-site-occupation case (*a*_Mg_ = 6.3477 Å). The calculation method and the results are detailed in Supplemental Material 3 in detail. We here summarize the results briefly. The calculation results showed that a shift of the site occupation of Ag occurred due to the restriction of the lattice constant; the *p*_Mg_ changed from 87% to 96% and the required pressure for inducing the shift was roughly estimated at 160 MPa. Although Mg_2_Si is known to be brittle, it can withstand high pressure and maintain its semiconducting properties under pressures of several gigapascals (GPa). Considering that a pressure of the order of tens to hundreds of MPa is usually applied during the synthesis of Mg_2_Si using the spark plasma sintering (SPS) method, it is presumably practical to apply additional pressure to shift the site occupation of dopants. Because of the page limitation, the calculation for other impurity species is described in Supplemental Material 3.

### 3.2. Substitutional impurities for hole doping

#### 3.2.1. Alkaline metals at Mg-site

Alkaline Li, Na, and K metals can serve as acceptors after replacing Mg atoms in the Mg_2_Si lattice, owing to the difference in their valences. Table 1 shows the lattice constants *a* of the optimized cells doped with Li, Na, and K atoms. Different changes in *a* are observed for various impurity sites, which can be explained by the differences in their covalent radii. When a Li atom occupies a Mg site, the larger covalent radius of Mg (139 pm) with respect to that of Li (133 pm) leads to crystal shrinkage; on the other hand, the Mg_2_Si crystal expands when Li replaces Si (116 pm). Similarly, doping with Na (155 pm) and K (196 pm) atoms results in larger lattice constants as compared to that of pure Mg_2_Si for all types of impurity sites.

From the optimized structures, the formation energies ΔE(*x*) were obtained (see Figure 3). The systems doped with Li for Mg and 4b sites exhibit relatively small values of ΔE (the substitution of its Si sites is very unlikely), which is in good agreement with the results of pseudo-potential first-principles calculations that were performed by Kolezynski et al. using a different computational software (USPPACK90). The Na- and K-doped systems with occupied Mg sites demonstrated the lowest ΔE; however, the interstitial insertion into 4b sites also resulted in small values of ΔE that were slightly higher than the values obtained for Mg sites. As indicated by the DOS depicted in Figure 3, the presence of these impurities causes hole doping in the case of Mg substitution, as was discussed earlier, whereas electron doping occurs when dopant atoms occupy 4b sites. Since the energies of Mg and 4b sites are almost the same, it can be suggested that doping with these impurities generates both holes and electrons.

The site-occupation probabilities of Li, Na, and K atoms are listed in Table 1. They exhibit finite values of $p_{\;4b}$, as well as $p_{Mg}$; in particular, the values of $p_{Mg}$ and $p_{4b}$ determined for Li insertion are comparable (55% and 45%, respectively). Although the obtained data do not support the stability of p-type conductivity, Li atoms can serve as potential acceptors in the Mg_2_Si lattice because of the remarkably low ΔE of Mg sites (such as –0.01 eV at *x* = 1.04 at.%). Moreover, as shown in Figure 3(a), the Li-related impurity states were different for Mg and 4b sites (in the former case, Li formed few states at the edge of the valence band, whereas in the latter case, distinct impurity states were produced near the edge of the conduction band). Thus, at low doping levels that are generally used experimentally (0.1–1.0 at.%), Li atoms substituted for Mg create a shallow impurity state, whereas doping 4b sites results in the formation of a deep localized state. This suggests a possibility that the number of holes produced by the Li substitution of Mg sites will exceed the number of electrons generated by interstitial Li (even when comparable numbers of Li atoms occupy the Mg and 4b sites), ultimately leading to p-type conductivity. The described calculation results can explain the experimental findings reported by Kolezynski et al. [15], who stated that Li-doped systems exhibited positive values of the Seebeck coefficient up to *x *= 3.0 corresponding to hole doping.

In addition, unusual behavior of the hole concentration was observed in the same work [15] (the Seebeck coefficient decreased with increasing Li content from *x *= 0.5 to 3.0), which could be attributed to the increase in the number of Li-occupied 4b sites, leading to electron doping. In fact, the X-ray diffraction data reported by this group revealed that the lattice constant *a* increased linearly with *x* according to the equation *a* = 0.004 *x *+ 0.6352 with *R* ^2^ = 0.98 [15] and were in good agreement with the calculation results obtained for 4b sites in this work (see Fig. 6(b) of Supplemental Material 1), which demonstrated a similar linear dependence (*a* = 0.0046 *x *+ 0.6348 with *R* ^2^ = 1.0) at *x* values below 4.167 at.%. Although Li is a potentially promising acceptor for Mg_2_Si because of its negative formation energy, the simultaneous occupation of 4b (n-type), as well as Mg (p-type) sites due to nearly the same formation energies pose a challenge in achieving stable p-type conduction. Application of adequate pressure can likely improve the controllability of the p-type conduction by Li doping, because it should increase the occupation of the Mg site by Li and suppress the occupation of other sites. This idea is discussed in Supplemental Material 3 in detail.

Finally, we discuss the solid solubility of these dopants. In the experimental work [16], it was reported that the solid solubility of Na in Mg_2_Si reaches a limit at *y* ≃ 1 for Mg_66.7-*y*_Si_33.3_Na*_y_*, which corresponds to *x *= 1.04% (i.e. Mg_63_Si_32_Na_1_) in the present paper. As shown in Table 2, the formation energy per Na atom, substituted for Mg, is given as 0.633 eV. Based on this result, it is assumed that the solubility limit of K is less than 1%, since it has a higher formation energy Δε, whereas the Li is expected to be substituted for Mg at *x *= 1% or more. We are not aware of comparative studies on the solubility limit of the Alkaline metals; however, an experimental work on MgSi_1−_ *_x_*Sn*_x_* [26] revealed that Li is a far more effective acceptor in comparison to Na or K because it exhibits better carrier activation and brings about a significant increase in the hole density. This experimental result is consistent with the foregoing prediction.

#### 3.2.2. Boron group elements at Si-site

The boron group elements B and Ga with a valence of 3 are expected to generate holes after their substitution for Si atoms characterized by a valence of 4.

The changes in *a* caused by incorporating these impurities are mostly consistent with the produced variations of atomic positions (see Table 1). For instance, the substitution of Mg and Si atoms with B decreases the value of *a*, owing to their attraction of the nearest-neighboring atoms. On the other hand, both the value of *a* and atomic positions remain nearly unchanged after the interstitial insertion of B into 4b sites.

The lowest formation energies were obtained for the doping of Si sites with B and Ga atoms (Figure 4). The B-doped systems exhibited relatively high formation energy, such as $\Delta \varepsilon_{Sisite}$ = 1.65 eV, which was one order of magnitude higher than the energy corresponding to the synthesis temperature of 1378 K equal to 0.119 eV (see Table 2). This result is unfavorable for B doping during the conventional synthesis process (especially when rhombohedral B crystals are used as the starting material, which was assumed when calculating the formation energies in Equations (1)–(3)). Selecting a suitable precursor with small formation energy is required for the successful fabrication of p-type B-doped Mg_2_Si. Here, we recall that the experimental solubility limit of Li at the Si site is approximately 1% [16], and in this case, our calculated formation energy per atom Δ*ɛ* is 0.633 eV. In contrast, B doping for the Si site exhibits a substantially higher Δε value of 1.650 eV, as shown in Table 2. Therefore, the solid solubility limit of B is likely to be less than 1 at.%.

As shown in Table 2, the Ga-doped system exhibits a value of $\Delta \varepsilon_{Si\;site}$ (0.067 eV) that is lower than the thermal energy corresponding to the synthesis temperature (0.119 eV); moreover, it is much lower compared with the B-doped system, which allows us to expect that the solid solubility limit of Ga is greater than that of B. The DOS obtained for this system (Figure 4(b)) is in good agreement with the results of a previous theoretical work conducted by Ihou-Mouko et al. [18], suggesting that its p-type conductivity could be successfully achieved via Ga doping. Indeed, their experimental measurements revealed that hole concentrations of 1.62 ×10^19^ cm^−3^ were achieved by the 0.8 at.% Ga doping [18]. Although close values of ΔE were determined for the Mg and Si sites, it would be difficult for Ga atoms to move from Si sites to other sites due to thermal fluctuations as they have a relatively large atomic mass. Consequently, it is expected that stable p-type conductivity can be maintained at elevated temperatures.

### 3.3. Acceptor candidates with high electronegativity

This section is devoted to the impurities with high electronegativity, such as F (3.98), Cl (3.16), and Br (2.96) [27], which can be potentially used as acceptors (for comparison, the electronegativities of Mg and Si atoms are equal to 1.31 and 1.90, respectively).

The lattice constants *a* of the MgSi_2_ systems doped with these impurities are listed in Table 1. According to the obtained results, doping with F atoms leads to lattice shrinkage; in contrast, doping with Cl and Br atoms expands it regardless of the type of occupied sites. The largest value of *a* was obtained for the Br-doped systems followed by the Cl- and F-doped ones, which was consistent with the order of their corresponding atomic radii Br > Cl > F.

As shown in Figure 5, the F- and Cl-doped systems exhibit the smallest values of ΔEfor 4b sites. Their corresponding DOS plots contain the Fermi levels in the valence bands, indicating that these impurities act as acceptors. In particular, the $\Delta E_{4b\;site}$ values obtained for the systems doped with Cl atoms at doping concentrations *x* below 2.08 at.% were almost zero, suggesting that Cl species easily occupied 4b sites. Although $\Delta E_{Si\;site}$ also exhibits a low value at *x *= 1.04 at.%, it increases with *x* more rapidly than $\Delta E_{4b\;site}$. Therefore, the formation energy per Cl atom calculated from the slope of the x−ΔE graph is different for the Si and 4b sites (according to Table 2, $\Delta \varepsilon^{4b}$= – 0.092 eV and $\Delta \varepsilon^{Si}=0.$125 eV), suggesting that the occupation of 4b sites is more energetically favorable. For the Br-doped system, the lowest ΔEvalue was obtained for Si sites followed by 4b and Mg sites. Since n-type conductivity is observed for the most stable Si sites (see the DOS depicted in Figure 5(c)), it is hard to achieve stable hole doping with Br atoms.

For doping with F, the lowest formation energy was obtained for the 4b site, as mentioned above, which suggests F insertion into the interstitial site. However, to elucidate the microscopic structure of the crystal doped with F, it may be necessary to examine the interaction between F and self-vacancies in Mg_2_Si. Experimental [28,29] and theoretical [30] studies have revealed that interstitial F in Si has a tendency to form complexes with vacancies. According to Ref. [30], the vacancy-F complexes have two ground-state configurations and they are negative-*U* systems that accompany lattice distortion. Therefore, in future studies, it is important to clarify whether F in Mg_2_Si also exhibits such behavior.

Finally, the structural changes observed after the addition of F, Cl, and Br atoms into the interstitial 4b sites must be discussed. First, we examine the opposite effects produced by F and Cl additions because they may provide important information on the volume of the interstitial region. As shown in Table 1 and Fig. 6 in Supplemental Material 1, the value of *a* decreased and increased with F and Cl doping, respectively. The atomic positions listed in Table 1 also demonstrate that the nearest-neighboring Mg atoms move towards interstitial F atoms and become repelled by the interstitial Cl species, which is consistent with the trends previously observed for the *a* values of these systems. Therefore, it can be concluded that the volume of the interstitial region around 4b sites is between the volumes occupied by F^–^ (the ionic radius: 136 pm) and Cl^–^ (181 pm) species. As shown in Table 1, moreover, the addition of Br atoms to 4b sites drives the nearest-neighbor Mg atoms away. As a result, the increase in *a* is larger than that obtained via Cl doping. Thus, the relatively high value of $\Delta E_{4b\;site}$ obtained for Br doping should be attributed to the structural instability of the produced system (in other words, it was difficult to dope 4b sites with Br due to its larger ionic radius of 195 pm).

## 4. Conclusions

Variable cell relaxation calculations were performed for the Mg_2_Si systems doped with several elements using the first-principles pseudo-potential method. As a result, the Ag-doped systems exhibited close values of formation energies and finite occupation probabilities for both 4b (n-type) and Mg (p-type) sites. The obtained results suggest a possibility of the simultaneous doping of holes and electrons during synthesis; moreover, the Ag atoms occupying Mg sites would be driven towards 4b sites at elevated temperatures. The same problem may be encountered for the systems incorporating alkali metals (Li, Na, and K) and Ga. However, Li needs small energy to occupy Mg sites; in addition, it forms shallow impurity levels adjacent to the valence band, which can be easily activated to dope holes. The possibility of using interstitial p-type dopants is also suggested in this work; it was found that Cl and F atoms tended to occupy the interstitial 4b sites leading to hole doping.

The obtained calculation results revealed that the lattice constant and atomic positions depended on the type of the sites occupied by an impurity atom, whose occupation characteristics could be controlled by varying the structural properties of the crystal. For instance, conducting the synthesis procedure at a relatively high pressure may prevent the insertion of Li and Ag atoms into 4b sites; in contrast, it would be favorable for the substitution of Mg sites with these atoms because the most stable structure obtained for 4b (Mg) sites has a larger (lesser) lattice constant as compared to that determined for pure Mg_2_Si.

In the present study, we limited our investigation to the three cases, namely, the substitutional Mg- and Si-site occupation and the interstitial insertion, as structures of a system incorporating impurities and estimated the occupation probabilities for these cases. However, to estimate the realization probability of these phases rigorously, more realistic simulations of the actual growth process are required; in particular, other side phases that can form from the Mg, Si, and dopant atoms need to be considered. If such side phases have a formation energy that is comparable to or lower than that of the impurity-doped Mg_2_Si, they will hinder the hole doping in the matrix. Furthermore, our calculation does not consider the anisotropic deformation of the cell through uniaxial pressing, which is generally used in SPS and other compaction methods. In order to consider the uniaxial pressing of a polycrystalline sample theoretically, various directions in which the pressure is applied to the crystal lattice should be taken into account. Although we have estimated the pressure necessary to realize a shift in the impurity occupation by assuming equiaxial deformation of the cell, further calculations for a uniaxially contracted cell are required to obtain a realistic guideline for actual material development. In addition, there are few experimental reports on Mg_2_Si doped with acceptors; in contrast, several experimental groups realized successful p-type doping for Mg_2_Si_1−_ *_x_*Sn*_x_* (0 <*x*≦1) (e.g. Ref. [31]), where Si atoms were partly replaced with Sn in the same crystallographic structure with Mg_2_Si. Therefore, more experimental studies on p-type Mg_2_Si are desired; further, comparisons of their doping ability with that of Mg_2_Si_1−_ *_x_*Sn*_x_* from the viewpoint of electronic and structural properties will enhance our understanding as well.
