# Supplementary material for: Substitutional and interstitial impurity p-type doping of thermoelectric Mg2Si: a theoretical study
Source: Sci Technol Adv Mater. 2019 Mar 14;20(1):160–72. doi: 10.1080/14686996.2019.1580537 (PMC6419642; doi:10.1080/14686996.2019.1580537)
Supplement: Supplemental Material [file TSTA_A_1580537_SM5307.docx]

# Supplemental Material 1: Dependence of the lattice constant on the impurity concentration

The dependences of the lattice constant *a* on the impurity concentration *x* are depicted in Fig. S1 for impurity-doped Mg_2_Si. The obtained data exhibit good linear characteristics for the majority of impurity species. As noted in Sec. 3.2.1, the data obtained at *x* ≦ 4.167 at.% for the interstitial insertion of Li were fitted using the method of least squares to achieve a better understanding of the anomalous behavior experimentally observed in Ref.[15]. As a result, the linear formula *a* = 0.0046 *x* + 0.6348 with *R*^2^ = 1.0 was obtained, which reproduced the corresponding XRD data [15] in the range of *x* from 0.5 to 3.0.

# Caption

Fig. S1. Dependences of the lattice constant *a* of the (a) Ag-, (b) Li-, (c) Na-, (d) K-, (e) B-, (f) Ga-, (g) F-, (h) Cl-, and (i) Br-doped Mg_2_Si on the doping concentration *x* (at.%) determined for Mg (circles), Si (triangles), and 4b (crosses) sites.

# Supplemental Material 2: Formation energy per impurity atom $\boldsymbol{\Delta}\boldsymbol{\varepsilon}_{\boldsymbol{i}}$ $\left( \boldsymbol{i}\mathbf{=Mg, Si, or 4b} \right)$

Figures 2‐5 in the main section show the formation energies $\Delta E$ (*x*) obtained for the conventional unit cell depicted in Fig. 1. In order to determine the occupation probability of impurity sites$p_{i} \left( i=Mg, Si, or 4b \right)$ in Eqs. (4)−(6), the corresponding formation energies per impurity atom $\Delta\varepsilon^{i}$must be computed from the values of $\Delta E_{i}$ (*x*). In this work, their magnitudes were estimated from the slopes of the fitting lines for the corresponding $\Delta E_{i}$ (*x*) graphs using the method of least squares. In other words, the $\Delta E_{i}$ values were fitted with the formula $\Delta E_{i} \left( x \right)$=$E_{0}+s_{i}x$, where $s_{i}$ was the adjustable parameter, and the formation energy at *x* = 0% $E_{0}$ was set to zero. During fitting, the data obtained at *x* = 1.04 and 2.08 at.% were used because the $\Delta E_{i}(x)$ function deviated from linearity at higher *x*. Moreover, in most experimental studies, the value of *x* was generally of the order of one or less. As an example, the $\Delta E_{i}\left( x \right)$ plots constructed for the Ag-doped systems and corresponding fitting lines are shown in Fig. S2. The formation energy per impurity atom $\Delta\varepsilon_{i}$ was determined via the formula $\Delta\varepsilon_{i}$ $=s_{i}\times100/12$. The fitting results and $\Delta\varepsilon^{i}$ values obtained for various impurities are listed in Table SI.

# Caption

Fig. S2. Formation energy graphs fitted for the Mg (circles), Si (triangles), and 4b (crosses) sites of the Ag-doped system using the method of least squares and equation $\Delta E_{i}(x)$=$E_{0}+s_{i} x$ ($E_{0}$= 0) at values of *x* below 4.17%. The calculated data points exhibit good linearity. The fitting results are listed in Table III.

Table SI. Fitting results of the $\Delta E_{i}\left( x \right) \left( i=Mg, Si, or 4b \right)$ graphs obtained for various impurity atoms using the method of least squares. The values of *x* were below 4.17%, and the fitting formula $\Delta E_{i}(x)$=$E_{0}+s_{i} x$ with $E_{0}$= 0 and the adjustable parameter $s_{i}$ was utilized. The obtained magnitudes of $s_{i}$ were converted to values per impurity atom to produce $\Delta\varepsilon_{i}$ ($\Delta\varepsilon_{i}=s_{i}\times100/12)$. The fitting errors and corresponding *R* values are listed as well, indicating that the obtained $\Delta E_{i}(x)$ graphs demonstrate linear dependences in most cases within the fitting range.

**Table SI.**

| Impurity | Occupation site  (conductivity type)  *i* | Slope $s_{i}$obtained by fitting $\Delta E_{i}$values | Fitting error of $s_{i}$ (eV) | R value | Formation energy per atom $\Delta\varepsilon_{i}$ (eV) |
| --- | --- | --- | --- | --- | --- |
| Ag | Mg site (p-type) | 0.063 | 0.002 | 0.9978 | 0.525 |
|  | Si site (n-type) | 0.120 | 0.001 | 0.9997 | 1.000 |
|  | 4b site (n-type) | 0.081 | 0.001 | 0.9995 | 0.675 |
| Li | Mg site (p-type) | -0.001 | 0.003 | 0.4343 | -0.008 |
|  | Si site (n-type) | 0.274 | 0.001 | 0.9999 | 2.283 |
|  | 4b site (n-type) | -0.008 | 0.004 | 0.4322 | -0.067 |
| Na | Mg site (p-type) | 0.076 | 0.003 | 0.9970 | 0.633 |
|  | Si site (n-type) | 0.431 | 0.004 | 0.9998 | 3.592 |
|  | 4b site (n-type) | 0.088 | 0.005 | 0.9935 | 0.733 |
| K | Mg site (p-type) | 0.168 | 0.002 | 0.9998 | 1.400 |
|  | Si site (n-type) | 0.599 | 0.005 | 0.9998 | 4.992 |
|  | 4b site (n-type) | 0.186 | 0.004 | 0.9987 | 1.550 |
| B | Mg site (n-type) | 0.437 | 0.0005 | 1.0000 | 3.642 |
|  | Si site (p-type) | 0.198 | 0.002 | 0.9998 | 1.650 |
|  | 4b site (n-type) | 0.413 | 0.002 | 1.0000 | 3.442 |
| Ga | Mg site (n-type) | 0.086 | 0.002 | 0.9988 | 0.719 |
|  | Si site (p-type) | 0.065 | 0.001 | 0.9997 | 0.540 |
|  | 4b site (n-type) | 0.118 | 0.001 | 0.9861 | 0.982 |
| F | Mg site (n-type) | 0.617 | 0.003 | 0.9999 | 5.142 |
|  | Si site (n-type) | 0.456 | 0.006 | 0.9995 | 3.800 |
|  | 4b site (p-type) | 0.301 | 0.004 | 0.9995 | 2.508 |
| Cl | Mg site (n-type) | 0.208 | 0.0004 | 1.0000 | 1.733 |
|  | Si site (n-type) | 0.015 | 0.007 | 0.7724 | 0.125 |
|  | 4b site (p-type) | -0.011 | 0.003 | 0.8223 | -0.092 |
| Br | Mg site (n-type) | 0.283 | 0.00001 | 1.0000 | 2.354 |
|  | Si site (n-type) | 0.075 | 0.007 | 0.9826 | 0.621 |
|  | 4b site (p-type) | 0.137 | 0.002 | 0.9996 | 1.141 |

# Supplemental Material 3: Change in the formation energy induced by external pressures

# *Formation Energy Calculation with Fixed Lattice Constant*

# As mentioned in the main section, the site occupation of impurity atoms may be controlled by applying an external pressure during synthesis. To estimate the pressure-induced shift of formation energies $\boldsymbol{\Delta}\boldsymbol{\varepsilon}_{\boldsymbol{i}}$ (*i*=Mg, Si, and 4b sites), we calculated the electronic states of systems doped with impurity atoms at 1.04 at.% for the n-type sites, assuming the lattice constant is fixed at the value of the optimized p-type system, which is denoted by *a*_P_. From the obtained total energies $\boldsymbol{E'}$, we calculated the formation energies by the following equations instead of Eqs. (1)−(3) in the main section:

$\Delta{E'}_{Mg site}=E'\left( \mathrm{Mg}_{2-3x}\mathrm{Si}A_{3x} \right)+3xE\left( \mathrm{Mg} \right)-E\left( \mathrm{Mg}_{2}\mathrm{Si} \right)-3xE\left( A \right),$ (S1)

$\Delta{E'}_{Si site}=E'\left( \mathrm{Mg}_{2}\mathrm{Si}_{1-3x}A_{3x} \right)+3xE\left( \mathrm{Si} \right)-E\left( \mathrm{Mg}_{2}\mathrm{Si} \right)-3xE\left( A \right),$ (S2)

$\Delta{E'}_{4b site}=E'\left( \mathrm{Mg}_{2}\mathrm{Si}A_{3x} \right)-E\left( \mathrm{Mg}_{2}\mathrm{Si} \right)-3xE\left( A \right).$ (S3)

In Supplemental Material 2, we obtainedthe formation energy per impurity atom $\Delta\varepsilon_{i}$ by linear least squares fitting of $\Delta E_{i} \left( x \right)$ at values of *x* below 4.17%. For Eqs. (S1)−(S3), however, we determine $\Delta{\varepsilon^{'}}_{i}$ using only $\Delta E_{i}$ obtained at *x*=1.04 % via the formula $\Delta{\varepsilon^{'}}_{i}$ $=\Delta{E^{'}}_{i}\times100/12$. The obtained $\Delta{\varepsilon^{'}}_{i}$ as well as$p_{i}$ calculated using Eqs. (4)−(6) in the main section are listed in Table SII.

As shown in Table SII, significant shift of the site-occupation of impurities occurred due to the restriction of the lattice constant in most cases. The condition that *a*=*a*_P_ seems to be rather effective to prevent the n-type site occupation because the $p_{i}$ values of 100% were obtained for several dopants. In the following, we will discuss the obtained results in detail.

# *Ag-doped system*

A shift of the site-occupation of Ag occurred due to the restriction of the lattice constant; such that *p*_Mg_  changed from 87% to 96%, which is favorable for the realization of p-type conductivity. This is due to the different dependences of the lattice constant *a* on the doping concentration *x* for the p-type (Mg) and n-type (Si and 4b) sites; i.e., *a* obtained in the stable state decreases with increasing *x* for Mg-site occupation, but increases with *x* for the other cases (particularly, a substantial increase in *a* was observed for the 4b site case), as shown in Fig. S1(a). Therefore, by restricting *a* to *a*_Mg_, systems doped with Ag for the 4b site become unstable, which causes an increase in the $\Delta\varepsilon_{4b}$.

The above-mentioned result allows us to expect that application of pressure on the system during synthesis will improve the controllability of the p-type conduction by Ag doping. The qualitative prediction of pressure for the realization of suppression of the cell to achieve *a*_P_ is difficult; however, we roughly estimate it using the relation between the external pressure *P* and the bulk modulus *K, K*≅-*P*/(Δ*V*/*V*_0_), assuming that the volume change Δ*V* (*=V*-*V*_0_ with the initial unit-cell volume *V*_0_ and *V = a*_P_^3^) is sufficiently small. In the present calculation, we assume that the *V*_0_ is given as *a*_0_^3^, where *a*_0_  is the lattice constant of the pure Mg_2_Si crystal (*a*_0_ = 6.354 Å), and use the experimental value of *K* for Mg_2_Si ~53 GPa). As a result, we obtain Δ*V/V*_0_ = -0.0030 and *P* ≅ *-K*Δ*V/V*_0_ = -0.16 GPa using *a*_P_=*a*_Mg_ (6.348 Å) for Ag doping. For a more rigorous estimate, assuming *V*_0_ = *a*_4b_^3^ (*a*_4b_ = 6.3593 Å), we obtain Δ*V/V*_0_ = -0.0055 and *P* ≅ -0.29 GPa.

Note that the discussion herein is based on several approximations; in particular, we used the bulk modulus *P* observed for a bulk crystal to estimate the change in the cell volume with applied pressure during synthesis. Thus, we obtained an approximate qualitative prediction of the effect of external pressure on the site occupation of impurity atoms. However, the results in Table SII suggest that lattice constant changes of the order of 1% significantly affect the occupation of sites by Ag atoms. Therefore, pressure-induced enhancement of the p-type conduction is expected for this system.

*Li-doped system*

We focus on Li-doped system as an example of Mg-substituting dopants. The electronic states for systems doped with Li for the Si and 4b sites were obtained under the condition that *a*=*a*_Mg_ (6.3476 Å). From the results, the formation energies were calculated using Eqs. (S2) and (S3). The obtained $\Delta\varepsilon_{i}$ and $p_{i}$are shown in Table SII. Comparing the values in Table II in the main section and Table SII, the differences between $\Delta\varepsilon_{\mathrm{Mg}}$ and those for the other two sites become larger by restricting *a*; in particular, $\Delta\varepsilon_{4b}$ turns out to be positive. This is because of the different dependences of *a* on *x* for the p-type and n-type cases, similarly for doping with Ag. The required pressure for changing *a*_0_ to *a*_Mg_ is estimated as 0.16 GPa via the previously mentioned calculation.

*B- and Ga-doped systems*

We now discuss the effect of pressure on the site occupation of Si-substituting dopants (B and Ga). The values of $\Delta\varepsilon_{i}$ and $p_{i}$ remain almost unchanged by the restriction of the lattice constant to *a*_Si_. This is because the lattice constants of the first and second stable states (i.e., the Si-and Mg-site occupation, respectively) have similar dependences on *x* as shown in Fig. S1(e) and (f). That is, since *a*_Si_ ≃*a*_Mg_, fixing *a* to the value of *a*_Si_ does not facilitate the hole doping process for these systems.

*Cl- and Br-doped systems*

Here, we examine the structural restrictions for enhancing the interstitial site preference of Cl and Br. The electronic-state calculation for the systems doped with Cl and Br with *a*=*a*_P_ (*a*_P_=6.368 Å and 6.377 Å for Cl- and Br-doped systems, respectively) were performed. As a result, the values of $\Delta\varepsilon_{Si site}$ of these systems increased substantially by the structural restriction, which implies that the Si site occupation is no more stable, and the $p_{4b site}$ around 100% is thereby obtained.

To realize this, a system with a larger *a*_P_ than *a*_0_ should be fabricated; however, this may be difficult to achieve by adjusting the sintering pressure, because decrease in the pressure during synthesis may lower the density of the samples, leading to poor electric conductivity as well as severe brittleness. To contrive a method to expand cells, introducing iso-electric impurities or defects should be one of the possible ways to change the cell volume without influencing the conduction type.

# Caption

Table SII. Formation energies per impurity atom $\Delta\varepsilon$ and the occupation site probability *p,* which were obtained under the condition that the lattice constant *a* is fixed at the value of the p-type-site-occupation case *a*_P_. The $\Delta\varepsilon$ was calculated using Eqs. (S1)−(S3) instead of Eqs.(1) −(3) in the main section.

**Table SII.**

| Impurity | Occupation site | Formation energy per atom $\Delta\varepsilon$ obtained for *a*=*a*_P_ (eV) ($\Delta\varepsilon$ for optimized cell)^**^ | Site occupation probability *p* for *a*=*a*_P_  ($p$ for optimized cell)^**^ |
| --- | --- | --- | --- |
| Ag | Mg site (p-type;  *a*_P_=6.348 Å) | 0.525 eV | 96% (87 %) |
|  | Si site (n-type) | 0.978 eV (1.000 eV) | 1% (1 %) |
|  | 4b site (n-type) | 0.857 eV (0.675 eV) | 3% (12 %) |
| Li | Mg site (p-type; *a*_P_=6.348 Å) | -0.008 eV | 84% (55 %) |
|  | Si site (n-type) | 2.261 eV (2.283 eV) | 0% (0 %) |
|  | 4b site (n-type) | 0.099 eV (-0.067 eV) | 16% (45 %) |
| Na | Mg site (p-type; *a*_P_=6.358 Å) | 0.633 eV | 100% (82 %) |
|  | Si site (n-type) | 7.178 eV (3.592 eV) | 0% (0 %) |
|  | 4b site (n-type) | 2.376 eV (0.733 eV) | 0% (18 %) |
| K | Mg site (p-type;  *a*_P_=6.370 Å) | 1.400 eV | 100% (88 %) |
|  | Si site (n-type) | 3.987 eV (4.992 eV) | 0% (0 %) |
|  | 4b site (n-type) | 1.156 eV (1.550 eV) | 0% (12 %) |
| B | Mg site (n-type) | 4.186 eV (3.642 eV) | 0% (0 %) |
|  | Si site (p-type; *a*_P_=6.334 Å) | 1.650 eV | 100% (100 %) |
|  | 4b site (n-type) | 3.4340 eV (3.442 eV) | 0% (0 %) |
| Ga | Mg site (n-type) | 0.714 eV (0.719 eV) | 31% (30 %) |
|  | Si site (p-type;  *a*_P_=6.355 Å) | 0.540 eV | 69% (68 %) |
|  | 4b site (n-type) | 1.132 eV (0.982 eV) | 0% (2 %) |
| F | Mg site (n-type) | 5.868 eV (5.142 eV) | 0% (0 %) |
|  | Si site (n-type) | 3.694 eV (3.800 eV) | 0% (0 %) |
|  | 4b site (p-type;  *a*_P_=6.336 Å) | 2.508 eV | 100% (100 %) |
| Cl | Mg site (n-type) | 2.272 eV (1.733 eV) | 0% (0 %) |
|  | Si site (n-type) | 0.722 eV (0.125 eV) | 0% (14 %) |
|  | 4b site (p-type; *a*_P_=6.368 Å) | -0.092 eV | 100% (86 %) |
| Br | Mg site (n-type) | 2.874 eV (2.350 eV) | 0% (0 %) |
|  | Si site (n-type) | 1.741 eV (0.660 eV) | 1% (98 %) |
|  | 4b site (p-type;  *a*_P_=6.377 Å) | 1.150 eV | 99% (2 %) |

* Site occupation probability is calculated at synthesis temperature (1378 K).

** The data in the parentheses are the $\Delta\varepsilon$ or $p$ obtained for the optimized structures without restriction of the lattice constant. That is, the data in Table II in the main section are shown again for comparison with the results obtained for *a*=*a*_P_.
